# Correlations between Excessive Body Mass Index, Body Perception, Physical Activity, and Respiratory Functions among Youths in an Urban Setting of Vietnam

**DOI:** 10.1155/2020/9627605

**Published:** 2020-08-24

**Authors:** Lan. P. T. Nguyen, Bach X. Nguyen, Tam T. Ngo, Yen H. N. Nguyen, Hang T. Phan

**Affiliations:** ^1^Department of Basic Medicine, Hanoi University of Pharmacy, Hanoi 100000, Vietnam; ^2^VNU School of Medicine and Pharmacy, Vietnam National University, Hanoi 100000, Vietnam; ^3^Faculty of Health Sciences, Thang Long University, Hanoi 100000, Vietnam

## Abstract

Young adults are susceptible to overweight and obesity and their adverse outcomes. However, limited studies have been conducted to understand this health problem in Vietnamese youths. This study was conducted to examine the rate of overweight and obesity, as well as the relationship between this condition on body perception, physical activity, and respiratory function among young adults in Hanoi, Vietnam. We performed a cross-sectional survey with 367 students aged 18-25 years at the Hanoi University of Pharmacy from June 2017 to June 2018. The result showed that the rate of overweight and obesity in our sample was 16.6%. There were 55.7% of overweight/obese students having the misperception of their body image. Results of multivariate regression analysis showed that overweight/obesity increased nearly three times (OR = 2.8, 95% CI: 1.5-5.0) the ability to be active. Overweight/obese students with physical activity had a better respiratory function. To conclude, this study underlined the associations between overweight and obesity with physical activity, body image, and respiratory function in our young sample. Further longitudinal studies should be warranted to examine their causal relationships.

## 1. Introduction

Overweight and obesity have been increasingly common in young adults around the globe [1]. These people are at high risk of being overweight/obese due to significant lifestyle changes in the transition stage from adolescence to adulthood [2, 3]. In low- and middle-income countries, previous systematic reviews indicated that the prevalence of obesity ranged from 2.3% up to 12% [1], and the rate of overweight and obesity was found to reach 22% in the university students aged 18 to 25 years [4]. There is ample evidence to show that being overweight and obese affected academic productivity and social relationships in young people [5, 6]. This problem also increases the risk of cardiovascular disease, diabetes, and metabolic disorders, as well as impaired respiratory functions in adulthood. Previous studies have shown that weight gain and high body mass index (BMI) were linked to a decrease in lung volume, which led to a decrease in Forced Expiratory Volume in 1st second (FEV1), Forced Vital Capacity (FVC), and Forced Expiratory Volume during 1st second (ERV) and a slight decrease in Residual Volume (RV) [7, 8].

Physical activity was associated with overweight/obesity. Physically inactive people are more likely to gain weight and have a higher risk of obesity than those who are physically active [9, 10]. Recent studies show that university students are not sufficiently physically active as per he recommendations of the World Health Organization [11–13]. A survey in Norway showed that students in this country did less exercise than before, and the percentage of overweight increased significantly [11]. Conversely, physical activity and obesity constitute a vicious cycle when being overweight is also found to be a cause of reduced physical activity [14].

Excessive attention to the stigma and the pursuit of an ideal appearance can create negative feelings, leading to behavioral changes, which can lead to overweight or obesity [15]. Body perception, which reflects self-awareness, is a factor affecting how to deal with overweight and obesity [16]. Being aware of weight situation can also have a positive effect on promoting healthy weight control behaviors [17]. People who find that they are overweight tend to have weight control plans like exercise or increasing physical activity [18].

In Vietnam, overweight/obese has been an alarming phenomenon in recent years [19]. Some previous studies on obesity have been done in children and, more recently, among adolescents [20, 21]. However, there was a lack of studies on young people, such as university students. Therefore, this study was conducted to understand the relationship between overweight and obesity on body perception, physical activity, and respiratory function in young adults in Hanoi.

## 2. Materials and Methods

### 2.1. Study Design

This cross-sectional study was conducted on 367 students of the Hanoi University of Pharmacy from June 2017 to June 2018. Subjects were 18-24 years old and did not have the following exclusion criteria: (1) having chronic and acute nasopharyngeal disease and pulmonary bronchial disease; (2) having chest or respiratory muscle disease; (3) having spinal disease such as hunchback, scoliosis, and ankylosing spondylitis; (4) having cardiovascular disease (5) having fever or other acute illnesses; or (6) having surgery within the last six months.

### 2.2. Variables and Measurement

#### 2.2.1. Anthropometric Index

Students' height, weight, waist circumference, and bust size were measured at the beginning of the morning, and they are required to snack beforehand. They were invited into a measuring room, took off their shoes, and hung their coats and hats on a rack next to the scale. All participants were asked to get simple clothes without a hat and shoes before measuring. Each student's standing height with a ruler fixed on the wall, waist, and chest size with a soft tape measuring to an accuracy of 1 mm were measured. Weight was measured with a medical scale with a minimum division of 0.1 kg. Body mass index (BMI) was calculated according to the following formula:

(1) $$\begin{array}{c} \text{BMI}=\frac{\text{Weight }\left(\text{kg}\right)}{{\left[\text{standing height }\left(\text{cm}\right)\right]}^{2}}. \end{array}$$

Asian classifications of BMI were used with three levels: underweight (BMI < 18.5 kg/cm^2^), normal (18.5–22.9 kg/cm^2^), and overweight/obese (BMI ≥ 23 kg/cm^2^) [22].

The formula calculates Pignet index:

(2) $$\begin{array}{c} \text{Pignet}=\text{standing height}\left(cm\right)-\left[\text{weight}\left(kg\right)+\text{chest size}\left(cm\right)\right]. \end{array}$$

The formula calculates waist height ratio:

(3) $$\begin{array}{c} \text{Waist height ratio}=\frac{\text{waist}}{\text{standing height}}\;*100\;\left(\%\right). \end{array}$$

#### 2.2.2. Body Perception

We asked all students to rate their body perception to thin, regular, and fat levels. Then, we compared the measured and estimated BMI to determine body perception distortion. Students had accurate perception if their body perception was not different from the BMI classification (thin corresponds to being underweight, fat equals overweight, and obesity) and vice versa.

#### 2.2.3. Respiratory Function

The pulmonary ventilation functions were measured using the PowerLab system (ADInstruments, Australia). Students did not overeat or wear tight clothing and have a relaxing break 30 minutes before the measurement. Students were instructed and observed how to breathe before making precise measurements. The Tidal Volume (TV), the Inspiratory Reserve Volume (IRV), the ERV, the RV, the Vital Capacity (VC), the FVC, and the FEV1 were noted. We also calculate the Tiffeneau and Gaensler indices:

(4) $$\begin{array}{c} \text{Tiffeneau}=\frac{\text{FEV}1}{\text{VC}}, \end{array}$$

(5) $$\begin{array}{c} \text{Gaensler}=\frac{\text{FEV}1}{\text{FVC}}. \end{array}$$

#### 2.2.4. Physical Activity

The International Physical Activity Questionaire-Short Form (IPAQ-SF) with seven items used to assess the level of physical activity. Questions about how long they performed vigorous, moderate, mild physical activities, and sitting in the last week were used to calculate the amount of energy consumed for different levels of activity and the total amount of energy each week. The level of physical activity was divided into three types including low, medium, and high, according to the instructions of the questionnaire [23]. Also, we classified the participants into two groups: physically active and inactive. Active groups had the average level of physical activity for at least five days a week, on average 30 minutes or more per day, OR practiced physical activity at least three days a week, on average 20 minutes or more each day.

#### 2.2.5. Health Status and Behaviors

The Visual Analog Scale (VAS) was used for evaluating self-reported health status, with scores fluctuating from 0 to 100 (worst health to best health) [24]. Students were asked if they drank alcohol in the last six months and have ever been exposed to tobacco smoke (including active and passive smoking).

#### 2.2.6. Sociodemographic

We surveyed sociodemographic information, including age, gender, and living area.

### 2.3. Statistical Methods

Data were analyzed by Stata 14.0 software (Stata Corp LP, College Station, Texas, U.S.). The Chi-squared and Kruskal-Wallis tests were used to compare ratios and means between groups, respectively. We also use multivariate logistic regression models and multivariate linear regression models to understand the effects of overweight/obesity on body perception, physical activity, and respiratory function. In multivariate logistic regression models, the dependent variables were body perception awareness and level of physical activity (2 levels). FVC, FEV1, and Tiffeneau were dependent variables of the multivariate linear regression models. Statistical significance of *p* < 0.05 was accepted.

## 3. Results

The average age of the students was 20.2 ± 1.1 years in this study. Men accounted for 48.5% of the study, and 26.4% of the study subjects were from rural areas. The percentages of students drinking alcohol and smoking in the study were 21.3% and 90.7%, respectively. The rate of overweight/obese varied by age, gender, place of residence, and smoking status (including passive smoking) (*p* < 0.05) Table 1.

Respiratory function indicators such as TV, IRC, VC, and FEV1 indicators of overweight/obese subjects were significantly higher than those of others (*p* < 0.05).

Table 3 shows that students who were physically active at high levels have a higher rate of overweight/obese than underweight and moderate BMI. The time of vigorous physical activity and energy consumption was highest among overweight and obese students (*p* < 0.05). Respondents who were overweight/obese were more likely to have body misconception compared to other groups (*p* < 0.05) (Table 2).

Table 4 indicates that increasing one year of age would reduce the risk of misconceptions about body perceptions by 20%. Overweight/obese students were more likely to be physically active (OR = 2.8, 95% CI: 1.5-5.0) as well as have higher FVC (Coef = 0.4; 95% CI: 0.2-0.6) and FEV1 (Coef = 0.4; 95% CI: 0.1-0.6) than students with moderate BMI.

## 4. Discussion

We found a relatively high prevalence of overweight/obesity in our young samples. Moreover, the rate of people with a misperception of their body image was substantial. The results of this study also showed that being overweight/obese was associated with physical activity and respiratory function.

The study results found that 16.6% of young adults participating in the study were overweight/obese. This finding was lower than the average overweight/obese rate of students from 21 low- and middle-income countries, with 22.0% [4]. It is also lower than the overweight/obese rate reported in students in Asian countries like Thailand [25] and Malaysia (30.1%) [26]. The previous studies indicate that university students are at a high risk of weight gain due to lifestyle changes in the transitional age group [2, 3]. Our research shows that the rate of overweight/obesity in these university students was lower than the rate in secondary school students (11-14 years old) in Vietnam (21%) [21]. However, we did not have sufficient data on the participants' preuniversity weight in order to conclude that they had gained weight after being enrolled in the university. Further studies should be performed to compare the weight of youth people before and after university admission and identify their patterns of weight change. The results of the study are also consistent with previous documents showing that the rate of overweight/obese was higher in male students than in female ones [27], and in urban students compared with those from rural areas [28].

The result showed that body size perceptions were distorted across all BMI categories. There were 45.9% of overweight/obese people thinking that they were neither lean nor fat, and 9.8% of these people even felt thin. Several previous studies have reported that overweight/obese people often think their weight was lower than their actual weight [29]. Lemon et al. indicated that there were 14% of overweight women, and 25% of very obese men thought they were not overweight [30]. Another American study found that 23% of women and 48% of overweight men felt they were fit [31]. In another study in South Africa, the proportion of people aged 15 and older who had an inaccurate perception of body size was 84.5% [32]. Indeed, misperception about the weight condition can be intentional, which could be explained by the fact that social stigma associated with being overweight/obese was existing, affecting the body perception of overweight/obese people [30, 33]. Prior literature also showed that various reasons could affect a person's body perception such as normalizing the sight of being overweight and obese [34], using social networks [35], as well as other demographic characteristics, health behaviors, self-esteem, and social relationship factors [36, 37]. Body perception can significantly influence adult weight efforts [30, 32]. People who had accurate perceptions about their body image were more likely to have weight control plans [18] and promote healthy weight control behaviors [17]. Therefore, solutions to help young people to perceive their body accurately should be developed and promoted widely.

In this study, we could not find associations between smoking and hometown characteristics with body perception, physical activity, and respiratory. This result may be because almost our sample were exposed to tobacco smoke (i.e., active or passive smoking), which might lead to the homogeneity in our sample. Furthermore, during the study period, all of our samples lived in Hanoi—an urban setting; therefore, hometown origins might not have a sufficient effect on their behaviors or perceptions. On the other hand, alcohol drinkers were more likely to be physically active compared to nonalcohol drinkers. Previous studies found similar results, which might be since all participants were students who had a high likelihood of being exposed to alcohol, especially after sports events, ceremonies, or when they were dissatisfied with their body image [38].

Our finding showed that overweight/obese people had significantly higher energy expenditure per week than that of people with normal or underweight conditions. This phenomenon can be justified by that overweight/obese people performed physical activity to reduce their weight. However, the issue is that most of them perceived that they were not overweight or obese. Therefore, several reasons for this result could be assumed. Firstly, physical activity was measured by the IPAQ instrument with a recall period of seven days ago, which hindered the ability to capture the physical activity in a more extended period. Thus, there might be a chance that during the week before the interview, overweight/obesity participants participated in activities that required more energy consumptions compared to underweight/normal people. Secondly, this study used BMI in assessing overweight/obese. However, several previous studies showed that BMI's ability to assess overweight/obese still has limitations due to the failure to assess the amount of lean and fat in the participants' body [39]. In this study, there was also a chance that those being classified as overweight/obese were not overweight or obese but had a slim body shape, thanks to being physically active. Therefore, further study to examine the overweight and obesity status of youth people with more accurate approaches should be warranted.

Previous theories supported the view that obesity harmed the respiratory system. Increased weight and BMI are associated with a decrease in lung volume, which leads to a decrease in FEV1, FVC, and ERV and a slight decrease in RV [7, 8]. It was in contrast to the findings of our study. The overweight/obese people in this study had better respiratory function than those with normal and underweight conditions. This result was explained by the significantly higher level of physical activity among overweight/obesity groups compared to others. Therefore, it can be said that physical activity has an impact on the relationship between BMI and lung function. When analyzing the individual multivariate regression model in each active and inactive group, similar results were found in the active group. In the inactive group, there was no correlation between BMI classification and respiratory function. Previous studies have also demonstrated that physical activity, especially vigorous physical activity, may increase lung function parameters [40, 41]. Even so, a report by Steele et al. showed that the associations between obesity and respiratory function are independent of obesity and physical activity [42]. We propose to conduct additional longitudinal studies to examine the interactions and effects of physical activity and the relationship between obesity and respiratory function.

This study has revealed overweight/obesity and its impact on body perception, physical activity, and respiratory function. However, it still has drawbacks such as the lack of representation due to the limitations of convenient sampling, and the cause-effect relationship cannot be determined due to limitations of the cross-sectional descriptive study design.

## 5. Conclusions

This study underlined the associations between overweight and obesity with physical activity, body image, and respiratory function in our young sample. Further longitudinal studies should be warranted to examine their causal relationships.

## Figures and Tables

**Table 1:** Characteristics of research subjects.

Characteristics	Normal	Underweight	Overweight/obesity	*p*
	*n* (%)	*n* (%)	*n* (%)	
Total	222 (60.5)	84 (22.9)	61 (16.6)	
Gender				<0.01
Male	109 (61.2)	29 (16.3)	40 (22.5)	
Female	113 (59.8)	55 (29.1)	21 (11.1)	
Hometown				0.03
Urban	67 (69.1)	13 (13.4)	17 (17.5)	
Rural	155 (57.4)	71 (26.3)	44 (16.3)	
Exposing tobacco smoke (including active and passive smoking)				0.03
No	15 (44.1)	14 (41.2)	5 (14.7)	
Yes	207 (62.2)	70 (21)	56 (16.8)	
Alcohol drinkers				0.17
No	172 (59.5)	72 (24.9)	45 (15.6)	
Yes	50 (64.1)	12 (15.4)	16 (20.5)	
	Mean (SD)	Mean (SD)	Mean (SD)	*p*
Age (mean, SD - year)	20.4 (1.1)	20.0 (1.0)	19.8 (1.0)	<0.01

The average BMI, Pignet, and waist-height ratio of participants were 20.2 ± 2.3 (kg/cm^2^), 33.9 ± 10.6, and 43.2 ± 4.1%, respectively. The overweight/obese rate was 16.6%, and the underweight rate was 22.9% in the study.  Table 2 showed that the rate of misconception about body perception was 38.2%, which was statistically higher in overweight/obese students compared to students with moderate BMI and underweight (*p* < 0.05).

**Table 2:** Anthropometric index and body perception.

Characteristics	BMI classification			*p*
	Normal	Underweight	Overweight/obesity	
	Mean (SD)	Mean (SD)	Mean (SD)	
Anthropometric index				
Height (cm)	161.7 (8.3)	161.7 (8.3)	166.6 (8.5)	<0.01
Weight (kg)	45.6 (5.1)	45.6 (5.1)	68.0 (8.2)	<0.01
Waist (cm)	64.4 (4.3)	64.4 (4.3)	77.9 (12.4)	<0.01
BMI (kg/m^2^)	17.4 (0.9)	17.4 (0.9)	24.4 (1.6)	<0.01
Pignet	45.6 (5.3)	45.6 (5.3)	17.0 (10.8)	<0.01
Waist height ratio (%)	39.9 (2.6)	39.9 (2.6)	46.8 (7.4)	<0.01
Body perception (compared to BMI classification)	*n* (%)	*n* (%)	*n* (%)	*p*
Same	143 (64.4)	57 (67.9)	27 (44.3)	<0.01
Different	79 (35.6)	27 (32.1)	34 (55.7)	

**Table 3:** Physical activity characteristics.

Physical activity characteristic	BMI classification			*p*
	Normal	Underweight	Overweight/obesity	
	Mean (SD)	Mean (SD)	Mean (SD)	
Time for physical activity				
Vigorous physical activity time (minute)	62.9 (51.0)	49.7 (46.4)	76.2 (43.2)	0.01
Moderate physical activity time (minute)	75.0 (56.1)	75.6 (54.4)	84.3 (56.0)	0.51
Walking time (minute)	23.8 (17.1)	22.2 (14.2)	21.8 (12.4)	0.86
Sitting time (minute)	176.5 (19.1)	176.3 (21.9)	180.0 (-)	0.29
Energy consumption				
Vigorous physical activity per week (MET)	1367.7 (2072.1)	642.2 (709.2)	1637.6 (1716.4)	0.02
Moderate physical activity per week (MET)	1553.3 (2213.1)	1496.6 (2535.5)	1628.9 (1617.9)	0.32
Walking per week (MET)	379.8 (365.1)	366.5 (323.3)	367.3 (297.5)	0.87
Total energy consumption per week (MET)	3380.5 (2968.5)	2534.8 (2673.0)	3609.7 (2422.7)	0.12
Level of physical activity	*n* (%)	*n* (%)	*n* (%)	*p*
Low	102 (46.0)	35 (41.7)	21 (34.4)	0.03
Moderate	78 (35.1)	42 (50.0)	26 (42.6)	
High	42 (18.9)	7 (8.3)	14 (23.0)	
Type of physical activity				0.12
Inactive	140 (63.1)	56 (66.7)	31 (50.8)	
Active	82 (36.9)	28 (33.3)	30 (49.2)	

**Table 4:** The anthropometric index associated with body perception, physical activity, and respiratory function.

Anthropometric index	Body perception^a^ OR (95% CI)	Active^a^ OR (95% CI)	FVC^b^ Coef (95% CI)	FEV1^b^ Coef (95% CI)	Tiffeneau^c^ Coef (95% CI)
Gender					
Male	Ref	Ref	Ref	Ref	Ref
Female	1.1 (0.7; 1.9)	0.8 (0.5; 1.4)	-1.0^∗^ (-1.1; -0.8)	-0.8^∗^ (-1.0; -0.6)	0.1 (-0.1; 0.2)
Age	0.8^∗^ (0.6; 1.0)	0.9 (0.8; 1.2)	0.0 (-0.0; 0.1)	-0.0 (-0.1; 0.0)	
Hometown					
Urban	Ref	Ref	Ref	Ref	Ref
Rural	0.8 (0.4; 1.3)	0.8 (0.5; 1.3)	-0.1 (-0.3; 0.0)	-0.1 (-0.2; 0.1)	-0.0 (-0.1; 0.1)
Exposing tobacco smoke (including active and passive smoking)					
No	Ref	Ref	Ref	Ref	Ref
Yes	1.7 (0.7; 3.8)	2.0 (0.8; 4.8)	-0.1 (-0.3; 0.1)	-0.2 (-0.4; 0.1)	-0.1 (-0.3; 0.1)
Alcohol drinker					
No	Ref	Ref	Ref	Ref	Ref
Yes	1.8 (1.0; 3.2)	2.8^∗^ (1.5; 5.0)	0.0 (-0.1; 0.2)	-0.1 (-0.3; 0.1)	-0.0 (-0.2; 0.1)
Self-rated health (per 10.3 score)	0.9 (0.7; 1.2)	1.2 (0.9; 1.5)	-0.0 (-0.1; 0.0)	-0.0 (-0.1; 0.0)	-0.0 (-0.1; 0.0)
BMI classification					
Normal	Ref	Ref	Ref	Ref	Ref
Underweight	0.9 (0.5; 1.6)	0.8 (0.4; 1.5)	-0.1 (-0.3; 0.1)	-0.2 (-0.4; 0.0)	-0.0 (-0.2; 0.1)
Overweight/obesity	1.4 (0.6; 3.1)	3.6^∗^ (1.5; 8.4)	0.4^∗^ (0.2; 0.6)	0.4^∗^ (0.1; 0.6)	0.1 (-0.0; 0.3)
Waist (per 7.2 cm)	1.0 (0.7; 1.3)	0.8 (0.6; 1.1)	-0.0 (-0.1; 0.1)	-0.0 (-0.1; 0.1)	-0.0 (-0.1; 0.0)
Level of physical activity					
Low	Ref	Ref	Ref	Ref	Ref
Moderate	0.8 (0.5; 1.3)		0.0 (-0.1; 0.2)	-0.1 (-0.2; 0.1)	-0.0 (-0.2; 0.1)
High	0.6 (0.3; 1.3)		-0.1 (-0.3; 0.1)	-0.1 (-0.3; 0.1)	-0.0 (-0.1; 0.1)

^∗^ *p* < 0.05.

## Data Availability

Requests for access to individual subject data may be made to Bach X. Nguyen; please send an email to bachnx.smp@vnu.edu.vn.
